# Elemental Composition and Health Risk Assessment of Deep-Sea Teleost’s of the Levantine Basin

**DOI:** 10.1007/s12011-024-04298-y

**Published:** 2024-07-06

**Authors:** Nuray Çiftçi, Deniz Ayas

**Affiliations:** Faculty of Fisheries, Mersin University, Mersin, Turkey

**Keywords:** Deep sea, Teleost’s, Fish, Tissue metal(loid) level, Health Risk Assessment, Mersin Bay

## Abstract

The determination of metal(loid) (As, Fe, Al, Sr, Zn, Pb, Mn, Cu, Cr, and Cd) levels in the muscle tissue of 23 different deep-sea bony fish sampled off Mersin Bay (NE Levantine Basin) and the assessment of health risks for human consumption were aimed. Tissue metal(loid) concentrations were determined as dry weight and analyzed by inductively coupled plasma mass spectrometry (ICP-MS). The tissue metal(loid) concentrations (µg g dw) were converted to wet weight prior to health risk assessment calculations. Standard mathematical formulas were used to determine the health risk assessment. There was a statistically significant difference between the fish species in terms of tissue metal(loid) levels (*p* < 0.05). The highest metal(loid) level was found in *C. sloani* among other species. As and Fe had the highest and Cd the lowest tissue concentrations in the examined species (*p* < 0.05). The relationships between the metal(loid)s analyzed in the tissue were significant (*p* < 0.01;0.05). Fe had an antagonistic effect with Cd, while other metal(loid)s had a synergetic effect with each other. Risk assessment analyses were performed for the consumable species, and it was found that the estimated daily and weekly intakes were below the tolerable limits established by the Food and Agriculture Organization (FAO) and the World Health Organization (WHO). The target hazard quotient (THQ) values exceeded the threshold of 1 (THQ > 1) only for As. The target cancer risk (TCR) was below the tolerable limits (> 10^−5^) except for As, Cd, and Al.

## Introduction

Heavy metals and metaloids discharged into the seas as a result of anthropogenic activities are transmitted from the neritic zone to the oceanic zone. The long half-life of metal(loid)s and the continuity of discharge cause accumulation in marine species such as mussel [1, 2],crab [3, 4], fish [4],and macrophytes [5, 6] as a result of increasing the ambient concentration. Deep seas differ from coastal zones due to their unique hydrodynamic structure. The number of studies on the accumulation level of metal(loid)s transported to the open seas in deep-sea fish is quite limited due to the sampling difficulty.

The deep sea is a dark ecosystem with high pressure, low temperature, dissolved oxygen, and limited nutrients. In order to develop adaptation to these extreme environmental conditions, deep-sea fish have lower body mass index, older age at reproductive maturity, and longer life spans than coastal fish [7]. This adaptation allows them to use energy more effectively. The longevity of deep-sea fishes, which show high capacity to extreme conditions, suggests that they may have developed a strong detoxification ability against the chronic accumulation and toxic effects of metal(loid)s added as a result of natural and anthropogenic effects [8].

The Mediterranean Sea is under the influence of terrestrial and atmospheric inputs of metal(loid)s as a result of industrial and domestic wastes from surrounding countries [9]. The main pathways of dissolved metal(loid) inputs to the Mediterranean Sea are the atmosphere and rivers [10–16],underground seepage, volcanic activity, and shipping also play an important role [9, 17]. In the Levantine Basin, atmospheric input of Cu, Pb, Al, and Fe in the north and Cu, Pb, and Cd in the south have been reported to be high [18].

The Levant Basin allows the mixing of Atlantic Ocean waters entering the Mediterranean Sea through the Strait of Gibraltar. The salinity and density of the Atlantic waters, which circulate around the Mediterranean with the anticyclonic cycle and come to the Levant Basin, increase here due to evaporation. The dense surface waters settle to the bottom and return to the Atlantic Ocean with the downstream flow [19]. The mixing is important in terms of metal(loid) transport in the water column.

Above a certain concentrations of elements such as Cu, Zn, and Fe which have biological functions in animals, and also very low concentrations of elements such as Cd, Pb, and Hg not have biological functions cause metabolic and physiological changes in animal. Factors such as the interaction between metal(loid)s, species, diet, habitat, sex, age, and organization level are very important in the determination and interpretation of metal(loid) toxicity. Previous studies have addressed the effect of geographical area, sampling depth, feeding habits, and trophic position on metal(loid) accumulation in deep-sea organisms [7, 20–25].

Fish is one of the healthiest sources of protein for human consumption. Some of the species studied in this research consist of economically important species offered for human consumption. Metal(loid) s discharged into the oceans may pose a health risk due to the ingestion of consumable fish species by humans. Several methods have been established to assess the health risks that may result from the consumption of fish contaminated with metal(loid)s [26, 27].

The aim of this study was to determine the levels of Al, Cr, Mn, Fe, Cu, Zn, As, Pb, Cd, and Sr in the muscle tissue of 23 different deep-sea teleost fish species sampled from off the Levantine Basin and to assess the potential health risks associated with the consumption of species that consumed by people.

## Materials and Methods

### Sampling and Process of Metal(loid) Analysis

In this study, essential and non-essential metal and metalloid (As, Fe, Al, Sr, Zn, Pb, Mn, Cu, Cr, and Cd) levels were determined in the muscle tissues of 23 different deep-sea bony fish species (*Haplostethus mediterraneus*, *Gonostoma denudatum*, *Pagellus bogaraveo*, *Lepidotrigla dieuzeidei*, *Trachurus trachurus*, *Chlorophthalmus agassizi*, *Synchiropus phaeton*, *Coelorhynchus coelorhynchus*, *Phycis blennoides*, *Scorpaena notata*, *Helicolenus dactylopterus*, *Centracanthus cirrus*, *Anguilla aguilla*, *Nettostoma melanurum*, *Citharus linguatula*, *Lophius budegassa*, *Lepidopus caudatus*, *Macroramphosus scolopax*, *Peristedion cataphractum*, *Hymenocephalus italicus*, *Chauliodus sloani*, *Capros aper*, *Lampanyctus crocodilus*) sampled from the open waters of Mersin Bay in the Levantine Basin.

**Fig. 1:**
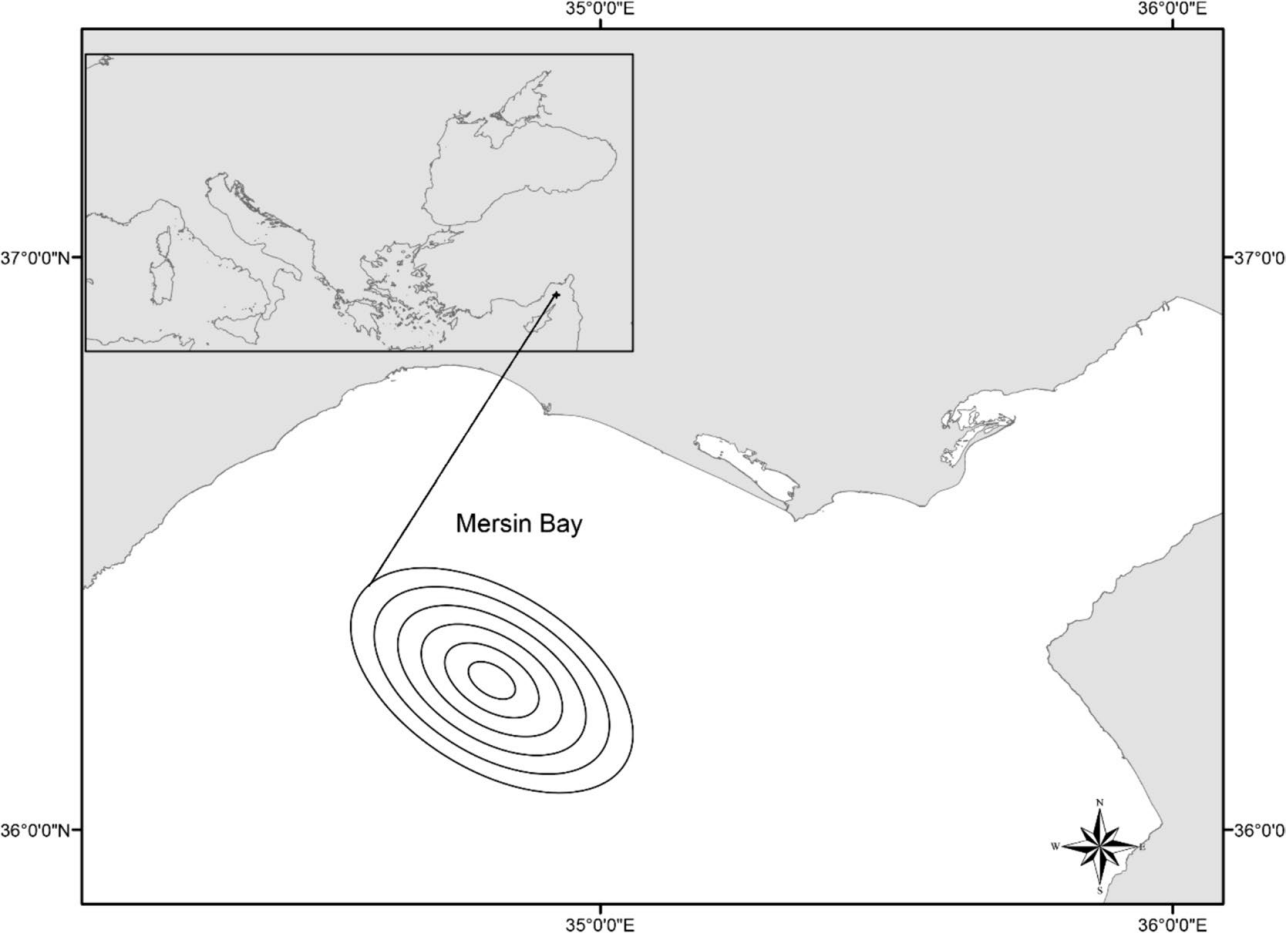
Map of the sampling area

Mersin Bay is one of the three gulfs located on the Turkish Mediterranean coast and is under the influence of a growing and developing industry due to the international port within its borders. Due to the port, the bay is under the influence of heavy maritime traffic. The dense population of Mersin province, which borders the bay, increases urban pressure. Agricultural activities, especially citrus, continue along the coast. The western shores of the bay, far from industry, are under the influence of tourism and nuclear power plant construction. The Göksu River is the main source of fresh water for the bay. The selection of the study area was based on the need to monitor the impact of coastal discharges from these sources on the open waters of Mersin Bay. The sampling area is presented in Fig. 1.

Between the 14th and 17th of May 2018, commercial trawls were used for deep-sea sampling in the international waters of Mersin Bay. The depth of the sampling area ranged from 274 to 641 m. The coordinates of the sampling area are as follows (Fig. 1): 36.24853N-34.36491E, 36.18839N-43.38847E, 36.17065N-34.40686E, and 36.07227N-34.53326E. The sampling was done for one period. It was carried out in six trawling operations with bottom trawls. The duration of each trawl operation was approximately 4 h. During the sampling process, 23 species of deep-sea fish were caught. The number of sampled species is presented in Table 3. The samples were brought to the laboratory in an ice box, and after measuring their length and weight, the dissected muscle tissues were dried in petri dishes at 95 °C for 72 h. Then, 0.1 g dried tissue was weighed and transferred to test tubes, and 2 ml of nitric acid (HNO_3_, 65%, Merck) was added [28] and burned on a hot plate set at 120 °C for 8 h [29]. The incinerated tissues were transferred to falcon tubes, and the total volume was made up to 10 ml with bidistilled water. Inductively coupled plasma mass spectrometer (ICP-MS, Agilent, 7500ce Model, Japan) was used to determine metal(loid)s. Samples were analyzed in triplicate, and three samples of each species were used.

Tissue metal(loids) concentrations were determined by the dry weight method [29], and data were converted to wet weight [30] prior to health risk assessment calculations.

### Quality Control and Quality Assurance

ICP-MS operating conditions were the following: radio frequency (RF) (W), 1500; plasma gas flow rate (L min^−1^),15; auxiliary gas flow rate (L min^−1^), 1; carrier gas flow rate (L min^−1^), 1.1; spray chamber T (°C), 2; sample depth (mm), 8.6; sample introduction flow rate (ml min^−1^), 1; nebulizer pump (rps), 0.1; extract lens (V), 1.5. The levels of As, Fe, Al, Sr, Zn, Pb, Mn, Cu, Cr, and Cd in samples were detected as μg metal(loid) g^−1^ dry weight. High Purity Multi-Standard (Charleston, SC 29423) was used for the determination of the metal(loid) analyses. Standard solutions for calibration curves are prepared by dilutions of the trace elements and potentially toxic metal(loid)s. Solutions have been prepared for the toxic metal(loid)s that had a content of Al, As, Cd, Pb, and Sr in the range of 1–50 ppb (0.001 to 0.050 mg l^−1^), and for the other elements that had a content of Fe, Zn, and As in the range of 1–50 ppm (1 to 50 mg l^−1^). International Atomic Energy Agency (IAEA-436) reference material was used to follow the quality of the analytical process. IAEA-436 was analyzed for all elements. The certified value and observed value of the IAEA-436 reference material were compared. Replicate analysis of this reference material showed good accuracy (Table 1). Six standards have been prepared for each metal. Validation parameters of the analytical method are given in Table 2.

**Table 1:** The certificated value and observed value of reference material IAEA-436

Analyte	Certified value 95%	Confidence interval	Observed value
As	1.98 ± 0.20	1.925–2.035	2.001 ± 0.022
Al	3.06 ± 0.42	2.944–3.176	3.112 ± 0.132
Cd	0.0492 ± 0.0042	0.0480–0.0504	0.0495 ± 0.0051
Sr	0.530 ± 0.073	0.510–0.550	0.533 ± 0.019
Cr	0.194 ± 0.058	0.178–0.210	0.211 ± 0.009
Cu	1.74 ± 0.19	1.678–1.793	1.731 ± 0.044
Fe	88.0 ± 6.5	86.198–89.802	90.001 ± 0.189
Zn	18.0 ± 1.3	17.66–18.360	18.876 ± 0.066
Mn	0.222 ± 0.026	0.215–0.229	0.244 ± 0.007

**Table 2:** Validation parameters of the analytical method

Analyte	LOD (ng g^−1^)	LOQ (ng g^−1^)	RSDr %	Recovery %	*R*^2^
Al	1.26	3.98	2.45	99.90	0.9975
Cr	1.19	3.87	2.73	98.10	0.9997
Mn	0.21	0.69	0.44	94.59	0.9972
Fe	1.46	5.01	4.59	97.02	0.9989
Cu	0.73	2.14	3.07	96.22	0.9991
Zn	2.34	7.56	3.32	95.60	0.9987
As	1.22	2.59	1.91	98.17	0.9992
Pb	0.53	1.48	2.12	95.95	0.9988
Cd	0.59	1.76	1.64	95.12	0.9990
Sr	0.004	0.013	0.009	94.30	0.9959

### Health Risk Assessment

An assessment was made of the health risk that the levels of metal(loid)s in muscle tissue of the sampled fish may pose as a result of human consumption. For this purpose, the estimated daily intake (EDI) was calculated using the following mathematical formula [31].

$$EDI (\text{mg}/\text{kg}/\text{day})=\frac{Cm \times IR \times EF \times ED}{Bw \times AT}$$

Here *Cm* is the signified metal(loid) concentration in fish muscle, *IR* is the signified daily consumption amount of consumer, which is assumed to be 0.017 kg/person/day in Turkey according to the Turkish Statistical Institute [32], *EF* means exposure frequency which is assumed to be 365 day/year, *ED* stands for exposure time of consumer which is assumed to be 70 years, and *Bw* refers to body weight of consumer which is assumed to be 70 kg. *AT* is the average life span calculated as body weight multiplied by 365 days/year and refers to the exposure time of the consumer. The estimated weekly intake (*EWI*) was calculated as the *EDI* value multiplied by 7 days.

The potential risk to humans from the consumption of metal(loid)-contaminated fish was determined by calculating the target hazard coefficient (*THQ*). The following formula was used for this purpose [31].

$$THQ=\frac{EDI}{\text{Oral }RfD}$$

where *RfD* is the oral reference dose for each metal(loid). The *Oral RfD* is expressed as the maximum safe level of a heavy metal(loid) ingested orally by an average adult weighing 70 kg. The *RfD* values used for calculation in the present study were 0.04 mg/kg/day for Cu, 0.3 mg/kg/day for Zn, 0.7 mg/kg/day for Fe, 0.003 mg/kg/day for Cr, 0.0003 mg/kg/day for As, 0.001 mg/kg/day for Cd, 0.004 mg/kg/day for Pb, 1.00 mg/kg/day for Al, 0.14 mg/kg/day for Mn, and 0.6 mg/kg/day for Sr [33]. The *THQ* exceeding the *RfD* (> 1) determined for each metal(loid) indicates adverse health effects associated with exposure to contaminated food [31, 34, 35].

The hazard index (HI) is an environmental health risk assessment that measures the potential risk of non-cancer health effects from exposure to multiple metal(loid)s. It is calculated by summing the THQ of each metal(loid)s. An HI less than 1 indicates that no adverse health effects are expected, while an HI greater than 1 indicates potential health risks. The HI is also expressed as a “total target hazard quotient” (TTHQ). The following formula was used to calculate the hazard index [31, 36].

$$HI=\sum_{i=1}^{n}THQn$$

The target cancer risk was calculated using the following equation (Hossain et al., 2018; [31]).

$$TCR=EDI\times CSF$$

The CSF is known as the carcinogenic slope factor. The CSF has been reported to be 1.5 mg/kg/day for As, 15 mg/kg/day for Cd, 0.0085 mg/kg/day for Pb, and 0.5 mg/kg/day for Cr. According to [37], acceptable limits for cancer risk are defined as 10^−4^ > CR > 10^−6^. CR > 10^−4^ indicates a cancer risk to consumers. The acceptable limit for *TCR* is 10^−5^ [38].

### Statistical Analysis

Descriptive statistics were applied to the data on metal(loid) levels determined in the muscle tissues of the species studied, and it was determined that the data did not have a normal distribution according to the Shapiro–Wilk. The non-parametric Kruskal–Wallis test for k-independent samples was used to test the differences between the levels of ten elements in the muscle tissue of 23 fish species. Then post hoc comparisons between groups of element levels between species were performed by the non-parametric Mann–Whitney test for two independent samples using SPSS 22.0 software. Linear regression analysis was used to compare the relationships between tissue metal(loid) levels and species. The Pearson linear correlation results were considered statistically significant at *p* < 0.01, 0.05.

A multivariate statistical analysis (Non-metric multidimensional scaling analysis—NMDS) was performed using the statistical software Past4.06b. It is based on a Euclidean distance matrix constructed on the basis of the metal(loid) level recorded in all samples to emphasize the discrimination between species in terms of metal level. The relationship between metal(loid)s was analyzed in a two-dimensional NMDS coordination plot. The NMDS model was found to be successful with a Proxscall Normalized Row Stress = 0.08. To accurately represent the data, the final stress values should ideally be less than 10% and not greater than 30%. Metal(loid)s with significant correlation to the axes are circled. Ninety-five percent confidence ellipses are shown for each group. The similarity and distance index between species in terms of tissue metal(loid) concentrations were based on the Bray–Curtis distance matrix. The distance between species in terms of tissue concentration of all metal(loid)s by clustering analysis and also the similarity between species by Neighbor-joining clustering analysis for each metal(loid) is based on the Euclidean similarity matrix.

## Results

**Fig. 2:**
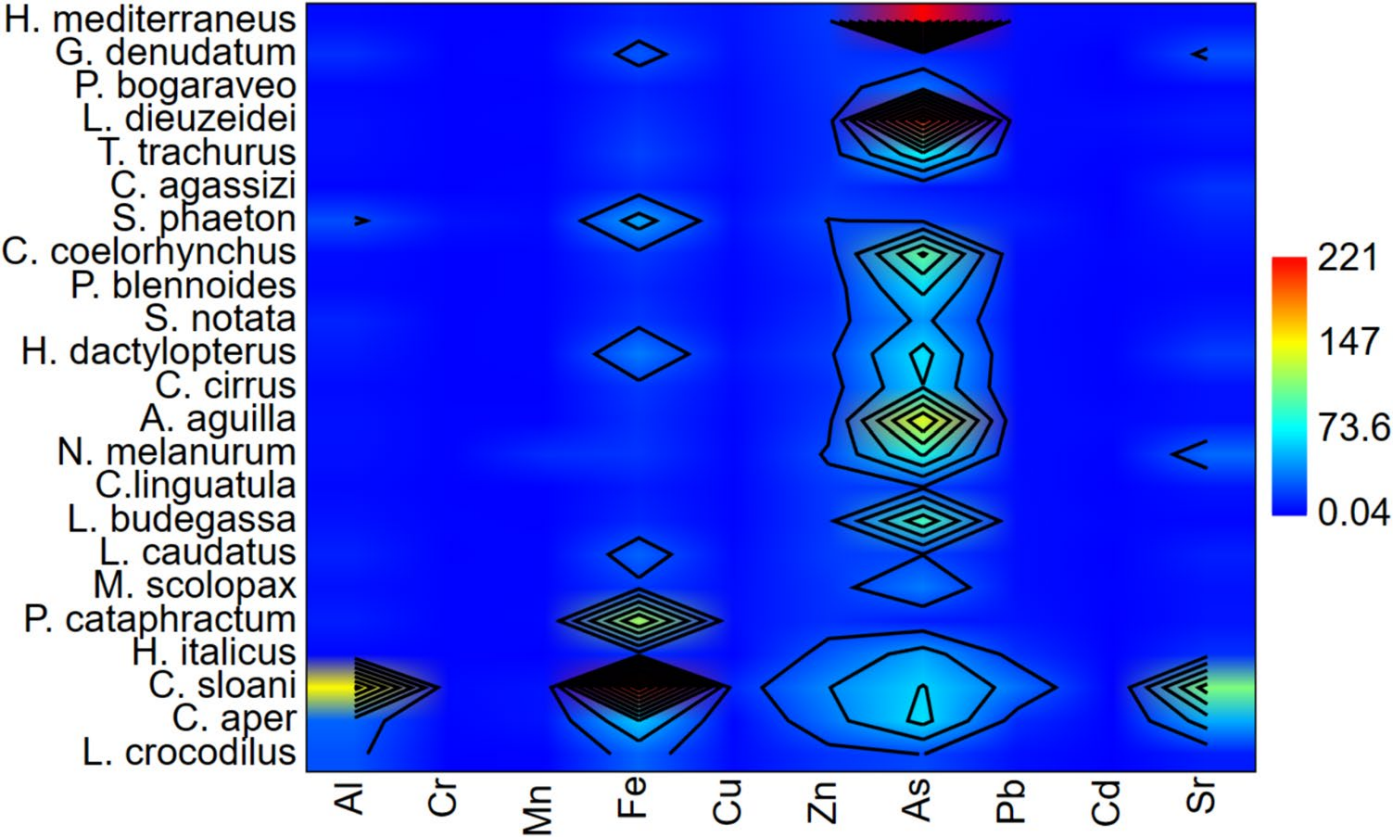
Matrix plot of species in terms of metal(loid)s level

The metal(loid) levels determined in the muscle tissue of 23 different deep-sea bony fish species sampled by bottom trawling off Mersin Bay are shown in Table 3. A statistically significant difference was found between the species in terms of tissue levels of metals and metalloids (*p* < 0.05). *C. sloani* had the highest tissue concentrations of the elements except As, Cr, Mn, and Cd. The highest tissue concentrations of As were found in *H. mediterraneus*, Cr in *S. phaeton*, Mn in *N. melanurum*, and Cd in *L. dieuzeidei* and *H. mediterraneus* (Fig. 2).

**Table 3:** Muscle tissue metal(loid) levels in deep-sea bony fish

Species	*n*	Al	Cr	Mn	Fe	Cu	Zn	As	Pb	Cd	Sr
		$\overline{x} \pm {s}_{x}$	$\overline{x} \pm {s}_{x}$	$\overline{x} \pm {s}_{x}$	$\overline{x} \pm {s}_{x}$	$\overline{x} \pm {s}_{x}$	$\overline{x} \pm {s}_{x}$	$\overline{x} \pm {s}_{x}$	$\overline{x} \pm {s}_{x}$	$\overline{x} \pm {s}_{x}$	$\overline{x} \pm {s}_{x}$
*Haplostethus mediterraneus*	25	3.09 ± 0.29^abst^	0.48 ± 0.32^as^	0.47 ± 0.04^as^	10.92 ± 0.45^abv^	5.01 ± 0.62^det^	15.62 ± 2.44^abcw^	220.61 ± 1.10^js^	3.29 ± 0.29^ast^	2.81 ± 0.22^ist^	4.56 ± 0.63^at^
*Gonostoma denudatum*	9	13.39 ± 6.11^cst^	0.50 ± 0.03^as^	1.44 ± 0.02^as^	26.12 ± 5.16^ct^	4.90 ± 0.87^des^	14.27 ± 1.12^abcst^	10.88 ± 2.12^at^	3.21 ± 0.26^as^	0.23 ± 0.07^abs^	23.31 ± 6.63^bct^
*Pagellus bogaraveo*	20 E	1.95 ± 0.13^abs^	0.43 ± 0.04^as^	0.41 ± 0.02^as^	9.06 ± 0.34^abt^	3.13 ± 0.13^abcdes^	13.72 ± 0.55^abcv^	32.10 ± 2.09^bcw^	2.95 ± 0.27^as^	0.47 ± 0.03^abcds^	2.36 ± 0.10^as^
*Lepidotrigla dieuzeidei*	30	3.97 ± 0.19^abs^	0.47 ± 0.02^as^	0.94 ± 0.02^as^	14.01 ± 1.11^abs^	2.95 ± 0.17^abcdes^	12.19 ± 1.02^abs^	206.69 ± 13.66^it^	3.17 ± 0.25^as^	2.99 ± 0.31^js^	7.70 ± 1.25^as^
*Trachurus trachurus*	22 E	3.72 ± 0.89^abs^	0.43 ± 0.02^as^	0.58 ± 0.01^as^	19.43 ± 1.12^bv^	3.86 ± 0.06^abcdes^	12.18 ± 0.69^abt^	75.09 ± 3.68^efw^	3.04 ± 0.26^as^	0.96 ± 0.04^defgs^	3.25 ± 0.65^as^
*Chlorophthalmus agassizi*	70 E	1.54 ± 0.67^as^	0.51 ± 0.08^as^	1.04 ± 0.18^as^	11.80 ± 1.23^abt^	2.14 ± 0.20^abcs^	14.64 ± 1.81^abct^	6.44 ± 0.56^as^	2.74 ± 0.13^as^	0.18 ± 0.04^abs^	14.54 ± 4.22^abt^
*Synchiropus sechellensis*	7	22.24 ± 1.67^dv^	4.86 ± 1.05^cst^	2.37 ± 0.25^ast^	48.18 ± 4.20^ew^	4.52 ± 0.35^cdet^	20.41 ± 2.02^bcdv^	18.75 ± 2.37^abv^	8.38 ± 0.91^bt^	0.35 ± 0.04^abcs^	8.85 ± 0.33^at^
*Coelorhynchus oelorhynchus*	2	3.21 ± 0.62^abs^	0.45 ± 0.03^as^	0.90 ± 0.20^as^	16.55 ± 2.52^abt^	2.30 ± 0.12^abcs^	14.11 ± 1.09^abct^	103.42 ± 2.68^gv^	3.51 ± 0.51^as^	1.42 ± 0.05^gs^	3.81 ± 0.41^as^
*Phycis blennoides*	10 E	2.71 ± 0.67^abs^	0.60 ± 0.04^as^	0.78 ± 0.06^as^	11.49 ± 2.91^abt^	1.70 ± 0.01^abs^	9.93 ± 0.29^at^	64.97 ± 3.93^defv^	3.00 ± 0.23^as^	0.84 ± 0.04^cdefs^	2.49 ± 0.25^as^
*Scorpaena notata*	23 E	10.07 ± 0.91^bctv^	0.46 ± 0.04^as^	1.05 ± 0.12^as^	15.81 ± 1.08^abv^	1.86 ± 0.13^abs^	13.00 ± 0.58^abyv^	44.28 ± 5.26^cw^	2.41 ± 0.12^as^	0.71 ± 0.11^bcdes^	7.26 ± 1.77^ast^
*Helicolenus dactylopterus*	20 E	6.37 ± 1.59^abs^	1.50 ± 0.48^as^	1.09 ± 0.16^as^	36.59 ± 2.13^dv^	5.15 ± 1.24^es^	17.35 ± 1.23^abct^	66.74 ± 2.52^defw^	2.54 ± 0.51^as^	1.09 ± 0.06^efgs^	17.62 ± 1.40^abt^
*Centracanthus cirrus*	3 E	2.99 ± 0.52^abs^	0.57 ± 0.05^as^	0.45 ± 0.04^as^	15.73 ± 2.28^abt^	2.63 ± 0.27^abcds^	12.79 ± 1.40^abt^	59.66 ± 1.57^dev^	3.02 ± 0.19^as^	0.87 ± 0.06^cdefs^	4.79 ± 0.46^as^
*Anguilla aguilla*	6 E	4.76 ± 0.64^abs^	0.86 ± 0.20^as^	0.88 ± 0.03^as^	13.04 ± 0.66^abt^	1.43 ± 0.04^abs^	16.01 ± 0.49^abct^	139.10 ± 2.27^hv^	2.93 ± 0.19^as^	1.90 ± 0.05^hs^	4.83 ± 0.30^as^
*Nettostoma melanurum*	2	4.21 ± 0.61^abs^	0.80 ± 0.09^as^	12.88 ± 2.00^ct^	15.04 ± 2.06^abt^	1.54 ± 0.27^abs^	21.74 ± 2.98^cdv^	82.34 ± 2.46^fgx^	2.89 ± 0.27^as^	1.16 ± 0.08^efgs^	30.79 ± 4.62^cw^
*Citharus linguatula*	35	2.74 ± 0.45^abs^	0.50 ± 0.02^as^	0.61 ± 0.07^at^	7.60 ± 0.51^at^	1.31 ± 0.06^abs^	16.08 ± 1.62^abcv^	18.75 ± 1.21^abx^	3.48 ± 0.27^as^	0.31 ± 0.01^abcs^	5.32 ± 0.54^aw^
*Lophius budegassa*	3 E	4.74 ± 0.39^abs^	0.85 ± 0.10^as^	0.58 ± 0.05^as^	10.78 ± 0.57^abs^	1.20 ± 0.04^as^	15.67 ± 0.80^abcs^	93.78 ± 12.64^get^	3.21 ± 0.89^as^	1.35 ± 0.23^fgs^	1.88 ± 0.10^as^
*Lepidopus caudatus*	8 E	9.02 ± 0.60^abct^	0.59 ± 0.01^as^	1.05 ± 0.06^as^	29.20 ± 1.68^cx^	2.53 ± 0.08^abcds^	16.84 ± 0.46^abcv^	19.67 ± 0.97^abw^	3.54 ± 0.32^as^	0.31 ± 0.00^abcs^	8.80 ± 1.82^at^
*Macroramphosus scolopax*	40	4.39 ± 0.25^abt^	0.91 ± 0.11^as^	1.06 ± 0.30^as^	16.31 ± 0.67^abv^	3.27 ± 0.96^abcdest^	14.28 ± 0.91^abcv^	35.96 ± 1.79^bcw^	3.55 ± 0.67^ast^	0.69 ± 0.25^bcdes^	5.20 ± 0.37^at^
*Peristedion cataphractum*	40	8.33 ± 0.87^abcv^	0.76 ± 0.12^as^	1.62 ± 0.16^ast^	119.93 ± 2.27^gx^	3.92 ± 0.99^bcdestv^	13.75 ± 1.75^abcw^	7.77 ± 1.02^av^	5.51 ± 0.03^abtv^	0.04 ± 0.01^as^	5.43 ± 0.19^atv^
*Hymenocephalus italicus*	15	2.04 ± 0.85^abs^	0.86 ± 0.20^as^	0.39 ± 0.15^as^	13.46 ± 2.52^abt^	1.58 ± 0.19^abs^	25.76 ± 1.58^dv^	48.42 ± 0.80^cdw^	13.73 ± 2.67^ct^	0.55 ± 0.12^abcds^	13.48 ± 3.18^abt^
*Chauliodus sloani*	4	148.10 ± 6.81^fy^	2.50 ± 0.63^bs^	5.21 ± 0.52^bs^	209.36 ± 4.91^ Hz^	11.65 ± 1.05^fst^	39.70 ± 5.40^fzw^	61.10 ± 4.33^dew^	33.28 ± 5.69^dtv^	0.36 ± 0.06^abcs^	110.60 ± 17.96^ex^
*Capros aper*	40	28.71 ± 1.38^ eV^	1.32 ± 0.34^as^	5.20 ± 1.03^bs^	58.42 ± 3.74^fy^	1.93 ± 0.43^abs^	33.36 ± 1.17^ eV^	65.35 ± 3.45^defz^	12.32 ± 2.28^ct^	0.84 ± 0.03^cdefs^	49.85 ± 2.03^dw^
*Lampanyctus crocodilus*	3	23.16 ± 2.99^dw^	0.59 ± 0.04^as^	0.70 ± 0.16^as^	28.29 ± 1.65^cx^	2.88 ± 0.38^abcdes^	14.69 ± 1.10^abcv^	20.32 ± 2.01^abw^	4.33 ± 0.27^ast^	0.42 ± 0.06^abcds^	8.02 ± 0.28^at^
Kruskall–Wallis											
*Chi-square*		57.922	50.9223	57.013	58.149	54.564	48.177	62.759	44.750	61.226	58.732
*Asymp. Sig*		0.000	0.000	0.000	0.000	0.000	0.001	0.000	0.003	0.000	0.000

The values with different letters (^a,b,c,d,e,f,g,h,i,j^) in the same row and the values with different letters (^s,t,v,w,x,y,z^) in the same column are significantly different (Mann–Whitney U test, *p* < 0.05)

$\overline{x} \pm {s}_{x}$: arithmetic mean $\pm$ standard deviation

The statistical distinction between metals and metalloids in terms of tissue concentrations was significant (*p* < 0.05). As, a metalloid, had the highest tissue concentration among the elements examined (220.61 ± 1.10 µg g^−1^ dw) followed by Fe (209.36 ± 4.91 µg g^−1^ dw), Al (148.10 ± 6.81 µg g^−1^ dw), Sr (110.60 ± 17. 96 µg g^−1^ dw), Zn (39.70 ± 5.40 µg g^−1^ dw), Pb (33.28 ± 5.69 µg g^−1^ dw), Mn (12.88 ± 2.00 µg g^−1^ dw), Cu (11.65 ± 1.05 µg g^−1^ dw), Cr (4.86 ± 1.05 µg g^−1^ dw), and Cd (2.99 ± 0.31 µg g^−1^ dw) (Table 3).

Figure 2 shows the similarity between species in terms of tissue metal(loid) levels. Color scale indicates tissue metal(loid) levels, and groups indicate species similar in accumulation level. Sr was found at the highest level in *C. sloani*. The similar species were *C. aper*, *N. melanurum*, and *G. denudatum*, respectively. In terms of Al level, *C. aper* and *L. crocodilus* were the species close to *C. sloani*, which had the highest tissue concentration. The average value shown on the scale was found to be dominant in the groups formed by the species showing closeness in terms of As tissue level. Fe was highest in *C. sloani* tissue, and the closest species was *P. cataprachtum*.

**Fig. 3:**
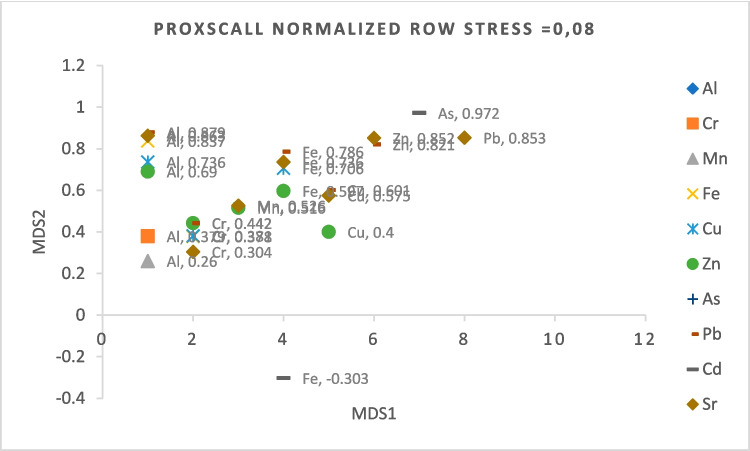
Multidimensional scaling of metal(loid)s in muscle tissue of deep-sea teleosts from offshore water of NE Mediterranean Sea

Table 4 shows the correlation between the metal(loid)s detected in the tissues. Fe was positively correlated with all metal(loid)s except Cd. Cd has a strong positive correlation with As (*r* = 0.972) and a weak negative correlation with Fe (*r* = 0.303) (Fig. 3). It was concluded that among the metal(loid)s found in the muscle tissues of the fish examined in this study, Fe was antagonistic with Cd and the interaction between other metal(loid)s was synergistic (*p* < 0.01; *p* < 0.05).

**Table 4:** Pearson correlation of metal(loid)s determined in muscle tissue of some deep-sea Teleost’s from offshore water of NE Mediterranean Sea

	Al	Cr	Mn	Fe	Cu	Zn	As	Pb	Cd	Sr
Al	1									
Cr	0.379** 0.002	1								
Mn	0.260* 0.036	0.172 0.170	1							
Fe	0.837** 0.000	0.378** 0.002	0.239 0.055	1						
Cu	0.736** 0.000	0.381** 0.002	0.059 0.641	0.706** 0.000	1					
Zn	0.690** 0.000	0.442** 0.000	0.516** 0.000	0.597** 0.000	0.400** 0.001	1				
As	− 0.094 0.454	− 0.162 0.196	0.000 0.994	− 0.202 0.106	0.033 0.794	− 0.058 0.647	1			
Pb	0.879** 0.000	0.442** 0.000	0.236 0.058	0.786** 0.000	0.601** 0.000	0.821** 0.000	− 0.086 0.498	1		
Cd	− 0.196 0.117	− 0.189 0.132	− 0.028 0.825	− 0.303* 0.014	− 0.042 0.736	− 0.134 0.287	0.972** 0.000	− 0.203 0.105	1	
Sr	0.863** 0.000	0.304* 0.014	0.526** 0.000	0.736** 0.000	0.575** 0.000	0.852** 0.000	− 0.061 0.632	0.853** 0.000	− 0.159 0.205	1

**Correlation is significant at *p* < 0.01

*Correlation is significant at *p* < 0.05

The multidimensional scaling plot was drawn to show the interaction between metal(loid)s. According to the graph, the correlation of Al with Pb, Sr, and Fe is stronger than the correlation with Cu, Zn, Cr, and Mn, respectively (*p* < 0.01). The correlation of Pb and Sr with Al, Zn, and Fe is stronger than with Cu and Mn, and the correlation coefficients are similar (*p* < 0.01; Fig. 3).

**Fig. 4:**
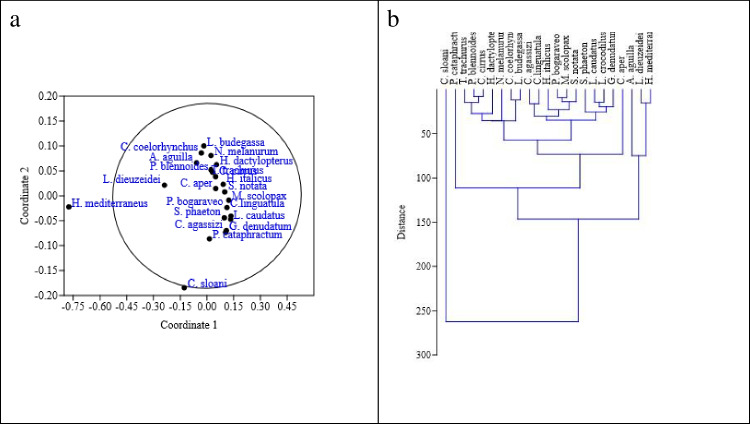
A**a** Similarities and distances index (Bray–Curtis). **b** Hierarchical clustering (Euclidean) of species in terms of muscle metal(loid) levels

The non-metric multidimensional scaling similarity index and scatter plot of the similarity among the deep-sea bony fishes analyzed in terms of tissue metal(loid) levels are shown in Fig. 4a, and the hierarchical clustering is shown in Fig. 4b. *C. sloani* and *H. mediterraneus* show a significant separation from other species in terms of tissue metal levels.

**Fig. 5:**
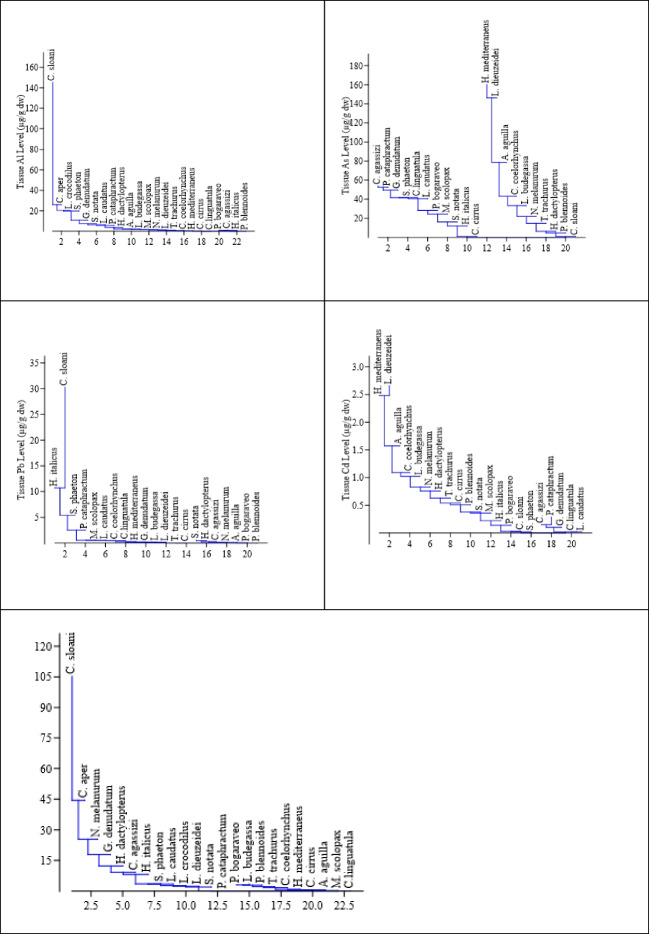
Clustering analysis of species for non-essential metal(loid)s level (Neighbor-joining clustering, Euclidean similarity)

Figure 5 shows the similarity of non-essential metal(loid)s in terms of tissue levels in the analyzed species. The toxic metal(loid)s Al and Pb were detected at the highest levels in *C. sloani* compared to the other species analyzed. In terms of Al level, *C. sloani* showed the closest similarity with *C. aper* with 32.47%. In terms of Pb level, *H. italicus* and *C. aper* showed the closest similarity with 58.41% and 54.04%, respectively. Cd and As, which are toxic metals and metalloids, were detected at high levels in *H. mediterraneus* and *L. dieuzeidei* tissues. In terms of Cd level, 96.90% similarity was found between these two species. In terms of As level, 96.74% similarity was determined between *H. mediterraneus* and *L. dieuzeidei*.

**Fig. 6:**
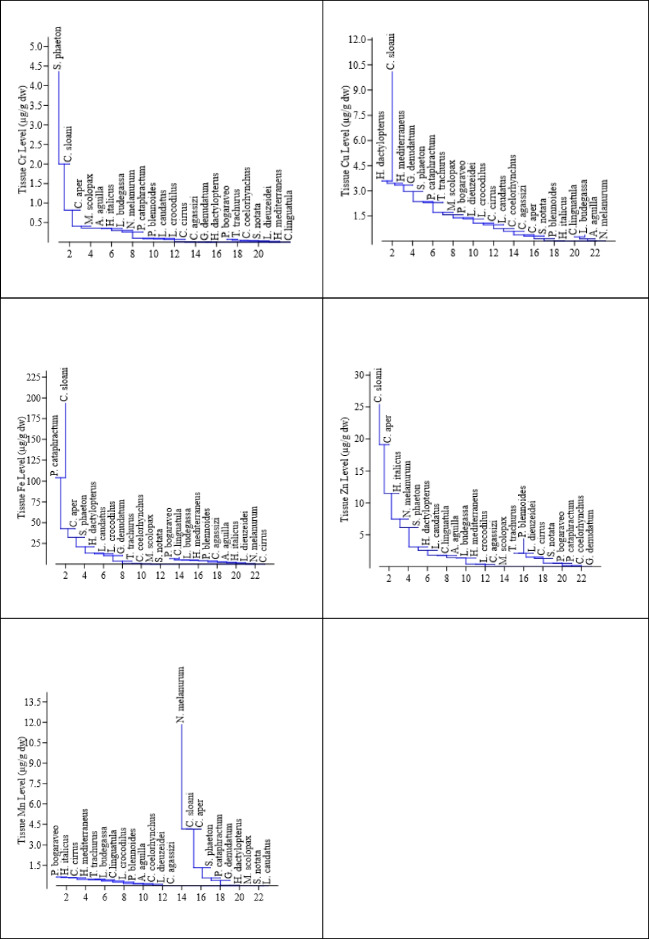
Clustering analysis of species for essential metal level (Neighbor-joining clustering, Euclidean similarity)

The highest tissue concentrations of the essential elements Cu, Fe, Zn, and Sr were found in *C. sloani* and showed similarity with the other species examined in the similarity analysis (Fig. 6). *C. sloani* was found in the same cluster with *P. cataphractum* (72.84%) in terms of tissue Fe level, *H. dactylopterus* (61.30%) in terms of Cu level, and C. aper (91.32% and 62.14% respectively) in terms of Zn and Sr levels. The highest Cr tissue level was found in *S. phaeton*, and *C. sloani* was the closest to this species with 67.93% in the similarity test. The highest level of Mn was found in *N. melanurum*, and it was in the same cluster with *C. sloani* (57.60%) and *C. aper* (57.52%) in the similarity test.

The estimated weekly intake in deep-sea fish was found to be below the maximum tolerable limit (EWI < 1; Table 5). The target hazard quotient for As was found to be above the tolerable limit in all species, and the hazard index was found to be above 1, depending on As (Table 6). In the species examined, the target cancer risk was below the acceptable limit of 10^−5^ except for As and Cd for all edible fish and Al only for *S. notata* and *H. dactylopterus* (Table 7).

**Table 5:** Estimated weekly intake (EWI μg/week/70 kg body weight) of metal(loid)s through consumption of the species with the corresponding potential tolerable weekly intake (PTWI μg/week/70 kg body weight)

	Estimated weekly intake (EWI)									
	Al	Cr	Mn	Fe	Cu	Zn	As	Pb	Cd	Sr
*P. bogaraveo*	0.0007	0.0002	0.0001	0.0032	0.0011	0.0049	0.0114	0.0010	0.0002	0.0008
*T. trachurus*	0.0013	0.0002	0.0002	0.0069	0.0014	0.0043	0.0266	0.0011	0.0003	0.0011
*C. agassizi*	0.0005	0.0002	0.0004	0.0042	0.0008	0.0052	0.0023	0.0010	0.0001	0.0051
*P. blennoides*	0.0010	0.0002	0.0003	0.0041	0.0006	0.0035	0.0230	0.0011	0.0003	0.0009
*S. notata*	0.0036	0.0002	0.0004	0.0056	0.0007	0.0046	0.0157	0.0009	0.0003	0.0026
*H. dactylopterus*	0.0023	0.0002	0.0004	0.0129	0.0018	0.0061	0.0236	0.0009	0.0004	0.0062
*C. cirrus*	0.0011	0.0002	0.0002	0.0056	0.0009	0.0045	0.0211	0.0011	0.0003	0.0017
*A. aguilla*	0.0017	0.0003	0.0003	0.0046	0.0005	0.0057	0.0492	0.0010	0.0007	0.0017
*L. budegassa*	0.0017	0.0003	0.0002	0.0038	0.0004	0.0055	0.0332	0.0011	0.0005	0.0007
*L. caudatus*	0.0032	0.0002	0.0004	0.0103	0.0009	0.0060	0.0070	0.0013	0.0001	0.0031
PTWI	490,000	1470	68,600	343,000	19,600	147,000	147	1960	490	294,000

**Table 6:** Target hazard quotients (THQ) of metal(loid)s through consumption of examined species with the corresponding hazard index

	Target hazard quotient (THQ)										HI	HI (except As)
	Al	Cr	Mn	Fe	Cu	Zn	As	Pb	Cd	Sr		
*P. bogaraveo*	9.90E-5	7.24E-3	1.50E-4	6.50E-4	3.95E-3	2.31E-3	5.41	1.00E-9	3.60E-7	1.99E-4	5.48	0.08
*T. trachurus*	1.88E-4	7.24E-3	2.10E-4	1.40E-3	4.87E-3	2.05E-3	12.64	1.00E-9	7.30E-7	2.74E-4	12.75	0.10
*C. agassizi*	7.80E-5	8.59E-3	3.80E-4	8.50E-4	2.70E-3	2.47E-3	1.08	1.00E-9	1.40E-7	1.22E-3	1.14	0.06
*P. blennoides*	1.37E-4	1.01E-2	2.80E-4	8.30E-4	2.15E-3	1.67E-3	10.94	1.00E-9	6.40E-7	2.10E-4	11.04	0.10
*S. notata*	5.09E-4	7.75E-3	3.80E-4	1.14E-3	2.35E-3	2.19E-3	7.46	1.00E-9	5.40E-7	6.11E-4	7.54	0.08
*H. dactylopterus*	3.22E-4	8.42E-3	3.90E-4	2.64E-3	6.50E-3	2.92E-3	11.24	1.00E-9	8.30E-7	1.48E-3	11.35	0.11
*C. cirrus*	1.51E-4	9.60E-3	1.60E-4	1.14E-3	3.32E-3	2.15E-3	10.05	1.00E-9	6.60E-7	4.03E-4	10.14	0.10
*A. aguilla*	2.40E-4	1.44E-2	3.20E-4	9.40E-4	1.81E-3	2.70E-3	23.42	1.00E-9	1.44E-6	4.07E-4	23.58	0.15
*L. budegassa*	2.39E-4	1.43E-2	2.21E-4	7.80E-4	1.52E-3	2.64E-3	15.79	1.00E-9	1.02E-6	1.58E-4	15.92	0.13
*L. caudatus*	4.56E-4	9.93E-3	3.80E-4	2.11E-3	3.20E-3	2.84E-3	3.31	2.00E-9	2.30E-7	7.41E-4	3.39	0.08

*HI* hazard ındex

**Table 7:** Target cancer risk (TCR) of metal(loid)s through concumption of species

	Target cancer risk (TCR)				
	Al	Cr	As	Pb	Cd
*P. bogaraveo*	8.00E-6	1.10E-5	**2.43E-3**	1.00E-6	**3.60E-4**
*T. trachurus*	1.50E-5	1.10E-5	**5.69E-3**	1.00E-6	**7.30E-4**
*C. agassizi*	6.00E-6	1.30E-5	**4.90E-4**	1.00E-6	**1.40E-4**
*P. blennoides*	1.10E-5	1.50E-5	**4.92E-3**	1.00E-6	**6.40E-4**
*S. notata*	**1.61E-4**	4.50E-5	**1.29E-2**	4.00E-6	**2.07E-3**
*H. dactylopterus*	**1.02E-4**	4.90E-5	**1.95E-2**	4.20E-6	**3.18E-3**
*C. cirrus*	1.20E-5	1.40E-5	**4.52E-3**	1.00E-6	**6.60E-4**
*A. aguilla*	2.00E-5	2.20E-5	**1.05E-2**	1.00E-6	**1.44E-3**
*L. budegassa*	2.00E-5	2.10E-5	**7.11E-3**	1.00E-6	**1.02E-3**
*L. caudatus*	3.80E-5	1.50E-5	**1.49E-3**	2.00E-6	**2.30E-4**

The values emphasised bold are above the acceptable limits for target cancer risk

## Discussion

In this research, the concentrations of some essential and toxic metals and metalloids in the muscle tissues of 23 different deep-sea fish species sampled off the coast of Mersin Bay were determined, and the relationships between the metal(loid)s in the tissue were interpreted in line with the data obtained. Accordingly, it was tried to make predictions about metal(loid) pollution in the deep seas, accumulation and detoxification of metal(loid)s in fish, and to evaluate the current situation of the open waters of the Northeastern Mediterranean, where limited information is available on this subject. Although sampling difficulties limit ecotoxicological studies in the deep sea, it is possible to find many studies on tissue metal(loid) accumulation in fish and other aquatic organisms in coastal ecosystems of the seas [28, 39–53] etc.).

Although the Earth’s crust contributes to metal(loid) concentrations in the oceans through natural cycles, it is supported by the literature that the metal(loid) contribution rate is greater as a result of anthropogenic factors [9, 54, 55]. The concentration of metal(loid)s added to the marine ecosystem varies depending on the physical properties of the environment such as temperature; light; currents and waves; chemical properties such as salinity, dissolved oxygen, conductivity, pH, phytoplankton biomass; and the amount of dissolved organic matter and suspended solids [56]. These factors play an effective role in the speciation of the metal(loid) added to the environment and in the formation of compounds with other metal(loid)s and cations and anions in the water. The complex structure that determines the metal(loid) concentration in the abiotic environment is maintained in biota under the influence of metabolic and physiological processes that play a role in metal(loid) uptake, accumulation, excretion, and detoxification. Therefore, metal(loid) toxicity varies depending on the species, organization level, developmental stage, sex, metal(loid), environmental concentration, interaction between metal(loid)s, and the abiotic factors that determine them. While metal(loid) accumulation and toxicity studies conducted under laboratory conditions are easier to interpret because a significant portion of the factors mentioned can be kept under control, it is more difficult to distinguish the variable that affects the result in studies conducted in nature. While the effect of a single metal(loid) can be studied in the laboratory, the interaction of metal(loid)s with each other and with physical and chemical changes in environmental factors, biological characteristics of the species, and its role in the trophic level should be interpreted together in natural studies where many metal(loid)s occur together.

This study attempted to interpret the levels of essential and toxic elements determined in the muscle tissues of bony fishes sampled by bottom trawl in Mersin Bay, representing the northeastern Mediterranean Sea, by considering the distribution, habitat, and diet of the fishes.

*C. sloani* was found to have the highest tissue concentrations of Al, Fe, Cu, Zn, Pb, and Sr among the species analyzed. The fact that *C. sloani* muscle tissue metal(loid) levels were higher than other fish species may be due to the fact that the species is a predator feeding on mesopelagic-benthopelagic fish and benthic invertebrates. Battaglia et al. [57] emphasized that *C. sloani* has a feeding strategy based on capturing small but large prey to maximize energy input, and cannibalism has been observed in this species. The knowledge that metal(loid) accumulates at higher levels in predatory species in the upper trophic zone may indicate the source of high metal(loid) levels in muscle tissue. One study suggested that the uptake and accumulation of lower concentrations of metal(loid) from the environment may have more toxic effects than the uptake and accumulation of higher concentrations from the diet. The detection of high levels of toxic elements such as Al and Pb in *C. sloani* muscle tissue may be the second indication in favor of dietary uptake. *C. sloani* is a species of Indo-Pacific origin. Indo-Pacific species are known to be highly adaptable. The fact that the species accumulated higher levels of metal(loid)s than the other species studied may indicate that the detoxification mechanism is strong. Mormede and Davies [25] stated that deep-sea species are expected to accumulate higher levels of heavy metal(loid)s than coastal species due to their longer life spans and trophic levels. In a study conducted in the southeastern Mediterranean Sea, it was reported that Hg and Cd levels were higher in crustaceans (Fe > Cu > Zn > Mn > Cd > Cd) and fish species (Fe > Zn > Mn > Cu > Hg ≥ Cd) sampled from the deep sea compared to coastal species, which may be due to dietary and physiological regulation of the metal(loid) [23].

Another reason for high metal(loid) accumulation may be the interaction between metal(loid)s. It is known that Zn, one of the bioessential elements, binds to 10% of all proteins in eukaryotic cells and acts as a cofactor, while less is found as cytosolic Zn, which is involved in neurotransmission and cellular signaling. Toxicological studies in fish show that the complexes formed by Zn with non-essential metal(loid)s are important in preventing toxicity. In this study, the synergy of Zn with essential and non-essential metal(loid)s other than Cd may be targeted to reduce toxicity by chelation. While Zn is involved in the structure of many proteins in animal organisms and is used in small amounts, Fe is an essential element involved in the structure of few proteins and is used in higher amounts. It plays an important role in the electron transport system, energy production, DNA, RNA, and protein synthesis in animal organisms. A total of 60–70% of Fe is found in hemoglobin and circulating erythrocytes and 10% in myoglobin. The remainder is stored in the liver and in macrophages of the reticuloendothelial system. The systemic balance of this element, which is toxic in excess, is provided by the control of absorption. In this study, Fe was negatively correlated with Cd and positively correlated with other elements except As and Mn. The presence of metal(loid)s in ionic form in the cell causes toxicity. Accordingly, the interaction between metal(loid)s may prevent the metal(loid)s from forming complexes and being in free ionic form, thus preventing toxicity. Cu, another essential element, is present as a prosthetic group in the structure of about 30 enzymes in animal organisms. Like Fe, Cu causes the formation of reactive oxygen species when it remains as a free ion during redox reactions to be converted to a useful form in the cell. Deficiency and excess of these elements are attempted to be controlled by homeostatic mechanisms. In this study, Cu was found to be higher in *C. sloani* compared to other species, which may be due to Cu-thioneins released for detoxification of non-essential elements such as Al and Pb, which are also present in high concentrations.

The non-essential elements with the highest tissue concentrations in *C. sloani* were Al, Pb, and Sr. In studies indicating that a significant proportion of incorporation is of atmospheric origin, Al concentrations were reported to be higher in surface waters of the eastern Mediterranean Sea than in waters of the western Mediterranean Sea and the Atlantic Ocean [10, 58]. Atmospheric Al has been reported to accumulate as dissolved Al at high concentrations in Mediterranean sediments [59]. Atmospheric particulate inputs of Pb, Cd, Cu, and Zn were reported to be two to three times higher than riverine inputs in studies conducted in the western Mediterranean [10, 11, 14]. Decreasing concentrations of Pb in the entire water column of the Ligurian Sea from 1983 to 1995 have been documented as a result of the gradual ban on the use of Pb additives in gasoline [60]. This information emphasizes the importance of the atmospheric contribution to metal(loid) accumulation in the deep sea. According to limited quantitative data, submarine volcanic seeps and submarine freshwater inputs in coastal areas also contribute [17]. Intensive industrial activities, port operations, and commercial maritime traffic through the Suez Canal in the eastern Mediterranean countries may be important metal(loid) inputs to the eastern basin. It is difficult to explain the presence of Al and Pb, which have no biological function, at very high concentrations in *C. sloani* tissues. Elevated Al concentrations in acidic waters of aquatic ecosystems are known to cause disruption of membrane permeability in gill epithelial cells of fish, resulting in disruption of osmoregulation and death of fish. Al toxicity to fish varies depending on the type of Al (cationic, neutral, anionic), pH, and complexing agents. Low pH causes Al to dissolve into the free ionic form, which is known to be toxic to fish. It has been reported that hydroxy aluminosilicate species formed by silicon with Al at pH 5 prevent acute toxicity by reducing Al accumulation [61]. The finding of high concentrations of Al in *C. gariepinus* muscle tissue sampled from the Olifants River and the Klein Olifants River, which are under the influence of domestic, industrial, and agricultural activities near the town of Bethal in South Africa [62], demonstrates that Al can be taken up by fish and accumulated in high concentrations in muscle tissue depending on environmental conditions. The limited number of studies conducted with deep-sea fish species does not allow comparison of the results of this study. The correlation of Al with Pb and other elements may explain the detoxification provided by metal(loid) complexes. The paucity of toxicological data on deep-sea species, which can adapt to extreme conditions, does not provide an opportunity for comparison. Among the edible fish species sampled offshore in the Arabian Sea, *Psenopsis cyanea* (highest Pb concentration 1179 μg/kg), *Chlorophthalmus corniger*, *Neoepinnula orientalis*, *Glyptophidium argentium*, and *Synagrops japonicus* have been reported to have Pb concentrations exceeding edible limits. Consumption of lead through food can cause neurological and hematopoietic disorders, and high levels can cause death (Ajeeshkumar et al. 2021). In this study, Pb was found to exceed the acceptable limits in the fish species studied (*p* < 0.05).

Sr, one of the non-essential elements, was found at higher levels in *C. sloani*, *C. aper*, *N. melanurum*, and *G. denudatum* among the species examined in this study compared to other species. Previous studies reported that waterborne Sr accumulated at high levels in the soft tissues of fish, but high tissue concentrations did not cause mortality [63]. Environmental factors have been reported to play an important role in fish uptake and accumulation. Chowdhury and Blust [64] emphasized that the main uptake route of Sr is the gills and the level of Sr accumulation in tissues varies depending on the Ca concentration in the environment. It is known that there is competition between metal(loid)s as well as Ca in the uptake of metal(loid)s through Ca channels in nature, where many metal(loid)s coexist. In this study, Sr was positively correlated with the analyzed metal(loid)s except As and Cd (*p* < 0.01, 0.05). The high concentrations of Sr together with non-essential elements such as Al and Pb can be explained by the discrimination between uptake pathways (Al and Pb may be more taken up by trophic transport). The interaction of Sr with essential elements such as Zn, Fe, Cu, Mn, and Cr may be a detoxification mechanism evolved to reduce the deleterious effects of Sr. Considering that the main uptake route of Sr is the gills, it is assumed that the tissue Sr level is related to the ambient concentration. The main source of Sr in nature is oil and gas extraction activities [65]. As a result of the exploration activities carried out in the last decade in the Levantine Basin, offshore areas of the Eastern Mediterranean coasts of Egypt, Cyprus, Israel, Palestine, and Egypt, many giant gas fields such as Zohr, Nargas, Shorouk, and Noor, have been discovered in Egypt. In addition, Cyprus announced another giant gas field, including Aphrodite, Israel started production from Leviathan, Karish, Athena, Dalit, and Tamar, and a marine gas field was discovered off the coast of Gaza [66]. Possible leakage from these activities may have caused an increase in Sr concentrations in the depths of the Levantine Basin. Species with high tissue concentrations sampled from the deep sea are thought to have a greater capacity to regulate these elements than coastal species.

As was identified as the element with the highest tissue concentration among the metal(loid)s analyzed and was found at the highest level among the elements analyzed in 16 (70%) of the species sampled in the study. Like many elements introduced into the oceans, As can be transformed into different chemical forms under the influence of the physical and chemical environment and can form complexes with organic and inorganic substances. Biomethylation of inorganic As, which is highly toxic to living organisms in high concentrations, into organoarsenicals allows 99% of As accumulated in fish and invertebrates to be stored [67]. Unlike other elements, the high levels of As in the muscle tissue of many deep-sea fish species can be explained by the conversion and storage of As uptake into arsenobetaine as a result of As metabolism in muscle tissue. However, it is very difficult to comment on which of the ingestion pathways of As, which is ranked 14th in the oceans, environment, or food, is dominant. Organic arsenicals are converted back to inorganic As species by microbial decomposition as a result of death in the sediment [67]. Sediment is the habitat where all kinds of elements added to the marine ecosystem settle after being suspended for a certain period of time. Therefore, bottom-dwelling macrobenthos and their predators are affected by high metal(loid) concentrations both through direct interaction with the environment and through food. The highest tissue concentration was found in *H. mediterraneus* followed by *L. dieuzeidei*. *H. mediterraneus* is a cosmopolitan species and lives on the muddy bottom of the oceans. The muddy bottom is the habitat with the highest metal(loid) concentrations in the sediment. This may be due to the high adsorption capacity due to the increased surface area of the decreasing particle size. Similarly, *L. dieuzeidei* is also a demersal species. Both species feed on macrobenthic invertebrates. In the study, the lowest tissue As levels were found in *C. agassizi* and *P. cataphractum*, which have similar habitats and diets. Metal(loid) toxicity is known to vary between individuals and with the duration and concentration of metal(loid) exposure. The low tissue As levels in *C. agassizi* and *P. cataphractum* can be explained by detoxification and excretion mechanisms, which depend on the species, individual, exposure concentration, and duration.

Among the non-essential elements analyzed, Cd was detected at the highest tissue level in *H. mediterraneus* and *L. dieuzeidei*. This may be due to the synergism of Cd with As. The fact that Cd levels were also low in *C. agassizi* and *P. cataphractum*, where As tissue concentrations were low, supports the interaction between metal(loid)s. Cd has been shown to cause kidney damage and is known to affect liver function. The acceptable limit of Cd in fish muscle tissue is 0.3 mg/kg, and in this study, Cd levels were found above the acceptable limits in all species except *G. denudatum*, *C. agassizi*, and *P. cataphractum*. Pb and Cd levels were reported to be below acceptable limits in ten different marine fish species sampled from the South Java and West Sumatra Oceans, Indonesia [68].

In this study, Mn tissue concentrations were found to be highest in *N. melanurum*. *C. sloani* and *C. aper* have the closest similarity to *N. melanurum* in terms of Mn tissue concentrations. It has been reported that Mn concentrations in the Mediterranean Sea are mostly of lithogenic origin. It has been emphasized that the high concentrations found in surface waters may be of atmospheric origin [69]. It has been reported that dissolved Mn concentrations in the ocean water column decrease with depth but increase in some mixing zones [70].

Cr is a functional element in carbohydrate, protein, and lipid metabolism in animal organisms, and its toxicity depends on the valence state of Cr in the compound [71, 72], Cr6^+^ is more toxic than Cr3^+^ [73–75]. In this study, the highest tissue concentration of Cr was found in *S. phaeton*. *S. phaeton* is a deep-sea demersal fish, and its prey consists of benthic invertebrates. Cr levels in deep-sea macrobenthic invertebrates have been found to be higher than fish tissue Cr levels [76]. The role of trophic transport in tissue Cr levels of *S. phaeton* feeding on macrobenthos may be high. In this study, a weak positive correlation of Cr with Al, Fe, Cu, Zn, and Sr was found.

Trophic transport plays an important role in the uptake and accumulation of metal(loid)s in aquatic organisms. Metal(loid) absorbed into the body is bound by metal-binding proteins such as metallothionein and tripeptides such as glutathione, synthesized in metabolically active tissues, to be detoxified and excreted from the body when it exceeds the carrying capacity of the tissues. The unexcreted portion is transferred to other tissues for storage. Therefore, metabolically active tissues accumulate metal(loid)s at a higher rate than inactive tissues. The amount of metal(loid) taken into the body by ingesting a metal-contaminated prey may be higher than that taken in by direct interaction with the environment. However, high concentrations of metal(loid) ingested through food are bound in biocellates, and elements released by absorption during digestion are incorporated into metabolic processes in the predator’s body. The direct effect of the ambient concentration may be weaker under normal natural conditions than in a contaminated environment. This is due to the fact that when the gills are exposed to the metal(loid) present in the environment, the gills increase the diffusion distance by causing an increase in mucus secretion, slowing down metabolic activities, increasing energy requirements, and limiting the uptake of the metal(loid) into the body by reducing the amount of water filtered through the gills.

Coastal ecosystems are hotspots under the direct influence of terrestrial sources, and the metal(loid) composition of the deep sea may vary depending on changing environmental conditions. The Mediterranean Sea, fed by Atlantic Ocean waters through the Strait of Gibraltar, is an intercontinental inland sea divided into eastern and western basins by the Tunisia–Sicily Threshold and is vulnerable to anthropogenic activities of coastal countries. Studies have shown that pollution caused by anthropogenic activities increases the concentration of metal(loid)s in Mediterranean surface waters and that these metal(loid)s can be found in the water column [77–80],sediments [81–83],and marine organisms [50, 69, 84, 85].

The atmospheric contribution of metal(loid)s has been found to be higher in the Mediterranean Sea than in the open oceans [10]. Studies have reported higher concentrations of Cu, Zn, and Cd in Mediterranean surface waters than in marine waters [69, 86]. The presence of Cd and Zn in the water column has been determined, and it has been emphasized that concentrations vary regionally [69]. Atmospheric uptake of Fe and Al increases concentrations in surface waters, while volcanic activity is the source of concentrations in the deep sea and sediments [58, 87].

The exchange of metal(loid)s in the water column depends on mixing and stratification. It has been reported that Cd and Zn concentrations, which increase with depth, are homogeneously distributed at a depth of 500 m [69]. It has been emphasized that lateral advection and vertical mixing are particularly effective in the distribution of bioavailable elements [88]. It has been reported that Al, Ni, and Cd increased in the Great Eastern Basin due to intense atmospheric participation, while Mn and Zn concentrations did not increase [10, 58, 89]. Metal(loid) concentrations in the water column of the eastern Mediterranean have been reported to vary due to organic complexation [90], biological utilization, and transport by currents [87, 91–94].

It has been reported that the elemental composition of the Mediterranean Sea has a dynamic structure compared to open oceans due to biogeochemical changes. The elemental composition determined in deep-sea bony fishes sampled from the eastern Mediterranean basin was found to be quite high compared to fishes sampled from open oceans. For example, it was reported that the levels of As, Cd, Cu, Pb, Hg, and Zn in muscle tissue of edible fish species (*Lophius piscatorius*, *Aphanopus carbo*, *Molva dyp terygia*, *Micromesistius poutassou*, and *Merluccius merluccius*) sampled from the continental slope of the Rockall Trough west of Scotland were within acceptable limits and similar to previous studies conducted on deep-sea fish [25]. Similarly, the elements analyzed in various marine fish species in the southern Java and western Sumatra oceans of Indonesia were reported to be below acceptable limits [68]. The high concentrations found in this study may be due to the higher proportion of atmospheric sources. This may be because metal(loid)s of atmospheric origin are more soluble in the sea [10]. Because it is known that metal(loid)s are absorbed into the body in the form of free ions. In this case, it can be assumed that the eastern Mediterranean basin has higher concentrations of metal(loid)s than the open oceans. On the other hand, the strong detoxification capacity of the species studied, their high concentration tolerance, as well as the high affinity of the metal(loid)ls to each other and the corresponding bioconcentration of the metal(loid) complexes formed may explain the high tissue concentrations. Nevertheless, it is important that the results obtained be supported by further physiological studies.

Some of the fish are *P. bogaraveo*, *T. trachurus*, *C. agassizi*, *P. blennoides*, *S. notata*, *H. dactylopterus*, *C. cirrus*, *A. aguilla*, *L. budegassa*, and *L. caudatus* examined in the study are edible. Therefore, human health risk was assessed in edible species. The EWI value in deep-sea Teleost’s sampled from the open waters of Mersin Bay was found to be below the FAO and WHO tolerable metal(loid) intake level (< 1). The target hazard quotient and hazard index were found to be above the tolerable limits, and a non-carcinogenic health risk was detected in the examined species depending on As. Consumption of P*. bogaraveo*, *T. trachurus*, *C. agassizi*, *P. blennoides*, *S. notata*, *H. dactylopterus*, *C. cirrus*, *A. aguilla*, *L. budegassa*, and *L. caudatus* may cause As and Cd-related cancer risk in humans. *S. notata* and *H. dactylopterus* may also cause Al-related cancer risk in humans who consume them.

It has been reported that there was no non-carcinogenic health risk (THQ < 1 and TTHQ < 1) in humans as a result of consumption of edible fish species sampled from the northeastern Mediterranean Sea (Antalya, Mersin, and Iskenderun Bay) [95]. While numerous studies support this finding [41, 96–98], there was a non-carcinogenic health risk, although limited (TTHQ = 1.31 for *Mullus barbatus*) [99]. Kosker [99] reported that consumption of some bony fish species sampled from the Northeastern Mediterranean has a cancer risk in humans for As, Cr, and Cd, but not for Pb, and the findings are compatible with our research findings.

## Conclusion

Tissue metal(loid) concentrations determined in deep-sea bony fishes of the Levantine Basin were found to be higher than predicted levels compared to coastal species [47, 50, 52, 100], [28, 101] etc. Although metal(loid) concentrations in the water column and seafloor of the Levant Basin, which are vulnerable to anthropogenic pollution pressures, vary regionally under the influence of mixing and the thermohaline layer, Mediterranean bottom waters are generally homogeneous in terms of temperature and salinity. The accumulation and toxicity of metal(loid)s in marine species may vary depending on the interaction between the abiotic environment and metal(loid)s in the deep seafloor, where many metal(loid)s coexist. In this study, the reasons for the higher-than-predicted accumulation of essential and non-essential elements in many deep-sea bony fish species can be explained as follows, in order of importance. (1) Higher metal(loid) regulation capacity of deep-sea fish species compared to coastal species; (2) the synergy of essential elements (Zn, Fe, Cu) with non-essential elements (Pb, Cd, Al, As), which may be a detoxification mechanism developed to inhibit the toxic effect of non-essential elements; (3) the accumulation of metal(loid) at high concentrations may have occurred mainly by trophic transport—since the direct uptake of metal(loid) from the environment is provided by the gills through Ca channels, the metal(loid) should be taken up in ionic form in this way. However, consumption of contaminated prey may result in simultaneous uptake of metal(loid)s in different forms, free and bound in the biota, and absorption of metal(loid)s released during digestion may lead to higher accumulation. This study is expected to contribute to the limited number of deep-sea ecotoxicological studies by determining the elemental composition of deep-sea bony fishes of the Levantine Basin.

The northeastern Mediterranean is under the influence of heavy metal(loid)s transported terrestrially and atmospherically. Therefore, it is important to assess and monitor the potential health risks of fish caught and consumed here. Evidence suggests that consumption of some edible deep-sea fish sampled off Mersin Bay has posed a cancer risk to humans related to As, Cd, and Al.

## Data Availability

No datasets were generated or analysed during the current study.
